# Editorial: Mitochondria in metabolic reprogramming and immune activation: the key gene and therapeutic target

**DOI:** 10.3389/fgene.2025.1686852

**Published:** 2025-09-01

**Authors:** Yongzheng Guo

**Affiliations:** Department of Cardiovascular Medicine, Cardiovascular Research Center, The First Affiliated Hospital of Chongqing Medical University, Chongqing, China

**Keywords:** mitochondria, inflammation, immunometabolic crosstalk, metabolic reprogramming, therapeutic targeting

## Introduction

Mitochondria are now widely recognized as regulatory hubs that extend far beyond their canonical role in energy production. Acting at the crossroads of metabolic adaptation, redox homeostasis, innate immune signaling, and cell fate determination, mitochondria influence both physiological processes and pathological states (Breda et al., 2019; Rocca et al., 2023). Their dysfunction has been implicated in a broad spectrum of human diseases, including autoimmunity, cardiovascular conditions, chronic inflammation, and cancer (Suomalainen and Nunnari, 2024). Understanding how mitochondrial metabolic remodeling intersects with immune activation is, therefore, essential for uncovering key molecular drivers and therapeutic targets. This Research Topic was designed to highlight such advances and foster new perspectives at the intersection of mitochondrial biology, immunology, and translational medicine.

This Research Topic brings together five original and review articles, each offering novel mechanistic insights and translational opportunities. Despite the diverse biological contexts they cover, ranging from autoimmune disease and myocardial infarction to periodontitis and cancer, they all converge on a unifying theme: mitochondria as orchestrators of immunometabolic crosstalk and potential targets for therapeutic intervention.

## Mitochondrial DNA and autoimmune diseases

Mitochondrial DNA (mtDNA) functions not only as a biomarker of mitochondrial health but also as a potent damage-associated molecular pattern (DAMP). Liu et al. employed bidirectional Mendelian randomization and clinical validation to demonstrate a causal association between decreased mtDNA copy number in peripheral blood and heightened susceptibility to autoimmune diseases such as Crohn’s disease, type 1 diabetes, and rheumatoid arthritis. These findings indicate that mitochondrial genome instability may directly contribute to immune dysregulation, highlighting mtDNA content as a potentially modifiable target for managing autoimmunity.

## Mitochondria at the intersection of aging, cell death, and tumor immunity

Wang et al. provided a comprehensive review of mitochondrial roles in immunosenescence, regulated cell death, and tumor immune evasion. The authors emphasized how mitochondrial metabolic shifts, reactive oxygen species generation, and mitochondrial outer membrane permeabilization influence apoptotic signaling and immune cell activation. Their synthesis underscores the therapeutic potential of mitochondria-targeted strategies for enhancing anti-tumor immunity and delaying age-associated immune dysfunction.

## Vitamin A–induced metabolic reprogramming in periodontitis

Cheng et al. investigated the role of vitamin A in modulating mitochondrial metabolism and macrophage polarization during chronic oral inflammation. They demonstrated that retinoids lead to metabolic rewiring of macrophages via the JAK–STAT pathway and reduce periodontitis-associated inflammation. This study highlights nutrient–mitochondria–immune crosstalk as a novel axis of immunometabolic regulation, providing a promising direction for the therapeutic modulation of inflammatory diseases.

## Mitochondrial regulation of post-infarction inflammation

Hou et al. conducted a bioinformatics-driven analysis to identify mitochondria-related genes governing immune cell infiltration and inflammatory responses following myocardial infarction. The authors’ findings revealed key regulatory nodes within mitochondrial metabolic pathways that are involved in immune cell recruitment and reparative remodeling of the ischemic myocardium. This integrative analysis bridges mitochondrial gene networks with immunopathology, demonstrating the potential of mitochondria-focused strategies in the treatment of cardiovascular disease.

## Therapeutic targeting of mitochondrial protease ClpP in cancer

Kong et al. reviewed the structure–function relationship and therapeutic relevance of the proteolytic subunit of caseinolytic mitochondrial matrix peptidase (ClpP), a pivotal regulator of mitochondrial proteostasis. The researchers summarized recent progress in the development of ClpP agonists that selectively disrupt mitochondrial homeostasis in cancer cells, leading to impaired oxidative phosphorylation and apoptosis. This work reinforces the emerging concept of mitochondria as druggable nodes for selective oncologic interventions (Wedam et al., 2023; Mukherjee et al., 2023).

Collectively, these contributions highlight the multifaceted roles of mitochondria in shaping disease-specific immune responses and emphasize their value as biomarkers and therapeutic targets. Future research should focus on delineating cell–type–specific mitochondrial signaling networks, using multi-omics approaches to map immunometabolic pathways, and developing combinatorial strategies that balance mitochondria for therapeutic gain. By bridging fundamental biology with translational insights, these studies collectively point toward a new generation of mitochondria-targeted diagnostics and interventions with broad relevance across immunological, metabolic, and cardiovascular disorders.

## References

[B1] Breda C. N. de S. Davanzo G. G. Basso P. J. Saraiva Câmara N. O. Moraes-Vieira P. M. M. (2019). Mitochondria as central hub of the immune system. Redox Biol. 26, 101255. 10.1016/j.redox.2019.101255 31247505 PMC6598836

[B2] Mukherjee S. Bhatti G. K. Chhabra R. Reddy P. H. Bhatti J. S. (2023). Targeting mitochondria as a potential therapeutic strategy against chemoresistance in cancer. Biomed. and Pharmacother. = Biomedecine and Pharmacother. 160, 114398. 10.1016/j.biopha.2023.114398 36773523

[B3] Rocca C. Soda T. De Francesco E. M. Fiorillo M. Moccia F. Viglietto G. (2023). Mitochondrial dysfunction at the crossroad of cardiovascular diseases and cancer. J. Transl. Med. 21, 635. 10.1186/s12967-023-04498-5 37726810 PMC10507834

[B4] Suomalainen A. Nunnari J. (2024). Mitochondria at the crossroads of health and disease. Cell 187 (11), 2601–2627. 10.1016/j.cell.2024.04.037 38788685

[B5] Wedam R. Greer Y. E. Wisniewski D. J. Weltz S. Kundu M. Voeller D. (2023). Targeting mitochondria with ClpP agonists as a novel therapeutic opportunity in breast cancer. Cancers 15 (7), 1936. 10.3390/cancers15071936 37046596 PMC10093243

